# Artificial Intelligence-Assisted Fetal Ultrasound in Low-Resource Settings: Opportunities, Challenges, and Future Directions

**DOI:** 10.7759/cureus.114172

**Published:** 2026-08-08

**Authors:** Priyanka Kumari, Rajkumar Meena

**Affiliations:** 1 Obstetrics and Gynaecology, ESIC (Employees' State Insurance Corporation) Hospital, Alwar, IND; 2 Radiodiagnosis, Children's Hospital of Eastern Ontario, Ottawa, CAN

**Keywords:** artificial intelligence, fetal ultrasound, low-resource settings, maternal health, prenatal diagnosis

## Abstract

Artificial intelligence (AI) has emerged as a promising strategy to improve access to fetal ultrasound in low-resource settings, where shortages of trained personnel, limited infrastructure, and unequal access to diagnostic imaging continue to compromise maternal and fetal healthcare. This narrative review synthesizes current evidence on AI-assisted fetal ultrasound, focusing on its clinical applications, implementation experience, challenges, and future directions. The reviewed evidence demonstrates that AI can support multiple stages of the fetal ultrasound pathway, including gestational age estimation, automated biometry and image quality assessment, structural anomaly detection, fetal cardiac and movement monitoring, image enhancement, and edge deployment for resource-constrained environments. These technologies have shown encouraging diagnostic performance and the potential to facilitate task-shifting by enabling non-specialist healthcare providers to acquire and interpret ultrasound examinations with greater accuracy and consistency. However, widespread implementation remains constrained by limited representation of low-resource populations in training datasets, insufficient prospective multicenter validation, infrastructure and connectivity limitations, workforce training requirements, and unresolved regulatory, ethical, and governance issues. Future research should emphasize the development of locally representative datasets, prospective multicentre validation, resource-efficient AI models, robust regulatory frameworks, and implementation studies that assess real-world feasibility. In addition, health economic evaluations, including cost-effectiveness analyses, are needed to determine the affordability and sustainability of AI-assisted fetal ultrasound in resource-constrained healthcare systems. With these advances, AI-assisted fetal ultrasound could play an important role in expanding equitable access to quality antenatal imaging and improving maternal and fetal healthcare in low-resource settings.

## Introduction and background

An ultrasound examination during pregnancy is one of the most cost-effective interventions in antenatal care, enabling gestational age (GA) dating, detection of multiple pregnancies, placental localization, and screening for fetal structural anomalies and growth disorders [1]. The World Health Organization recommends at least one ultrasound scan before 24 weeks of gestation for all pregnant women [2]. Despite this recommendation, access remains profoundly unequal: obstetric ultrasound services in many low- and middle-income countries are concentrated in urban referral centers, and rural or peripheral facilities frequently lack functioning equipment, reliable electricity, or personnel trained in image acquisition and interpretation [3,4]. Neonatal and perinatal disorders continue to account for a disproportionate share of global years of life lost, with the burden concentrated in sub-Saharan Africa and South Asia, underscoring the urgency of scalable diagnostic solutions [5].

Artificial intelligence (AI), and deep learning in particular, has matured rapidly over the past decade as a tool for automating image acquisition guidance, standard-plane recognition, biometric measurement, and anomaly detection in fetal ultrasound [6,7]. Early work in this field was explicitly motivated by the access gap in resource-limited settings. Yaqub et al. described one of the first deep-learning solutions for automatic verification of fetal neurosonographic diagnostic planes against clinical standard constraints, framing the underlying problem as one of assisting non-expert operators to acquire correctly standardized images [8]. Shortly afterward, van den Heuvel et al. demonstrated that fully convolutional neural networks could automatically detect the fetal head and estimate head circumference from free-hand obstetric sweep protocol data collected by minimally trained health workers in Wolisso, Ethiopia, explicitly designing the acquisition protocol so that it could be taught to any healthcare worker within a single day, without prior ultrasound experience; this study is widely regarded as establishing the technical and conceptual foundation for the blind-sweep AI paradigm subsequently scaled by later multicenter studies [9]. Contemporaneous work extended automated quality control to fetal cardiac four-chamber views and first-trimester three-dimensional volumetric assessment, while methodological advances in weakly supervised and self-supervised representation learning addressed the scarcity of expert-annotated training data that constrains model development in data-poor environments [10,11]. Unlike traditional ultrasound training, which requires years of supervised practice to achieve competency in probe manipulation and image interpretation, AI-enabled devices can, in principle, guide novice or minimally trained operators through a standardized acquisition protocol (commonly a “blind sweep”) and generate clinically actionable outputs without requiring an on-site expert [9,12]. This capability is particularly relevant to low-resource settings (LRS), where task-shifting of point-of-care ultrasound to midwives, nurses, and community health workers has already been shown to be feasible and to influence clinical management, albeit with important infrastructural and training barriers [4].

This narrative review aims to critically synthesize the evidence on AI-assisted fetal ultrasound in low-resource settings to provide a comprehensive overview of its clinical applications, implementation experiences, challenges, and future directions, while highlighting key evidence gaps and priorities for research and clinical practice.

## Review

Methodology

A comprehensive literature search was conducted using PubMed/MEDLINE, Scopus, and Google Scholar, supplemented by manual screening of the reference lists of relevant articles. The search included studies published between January 2017 and July 2026 and combined controlled vocabulary and free-text terms related to three key concepts: (i) artificial intelligence, machine learning, deep learning, or convolutional neural networks; (ii) fetal ultrasound, prenatal ultrasonography, or obstetric imaging; and (iii) low-resource settings, low- and middle-income countries, or resource-limited settings.

Eligible studies included peer-reviewed original research articles, review articles, study protocols, and guidelines published in English that described artificial intelligence, machine learning, or deep learning applications in fetal or obstetric ultrasound or wearable fetal monitoring, with a particular focus on their application, validation, feasibility, or relevance to low-resource settings. Studies conducted exclusively in high-income, well-resourced settings without discussion of applicability to resource-limited environments, conference abstracts, editorials, non-peer-reviewed preprints, and studies unrelated to obstetric ultrasound were excluded. Titles and abstracts were screened for relevance, followed by full-text assessment of potentially eligible articles.

The burden of limited access to fetal ultrasound in low-resource settings

**Figure 1:**
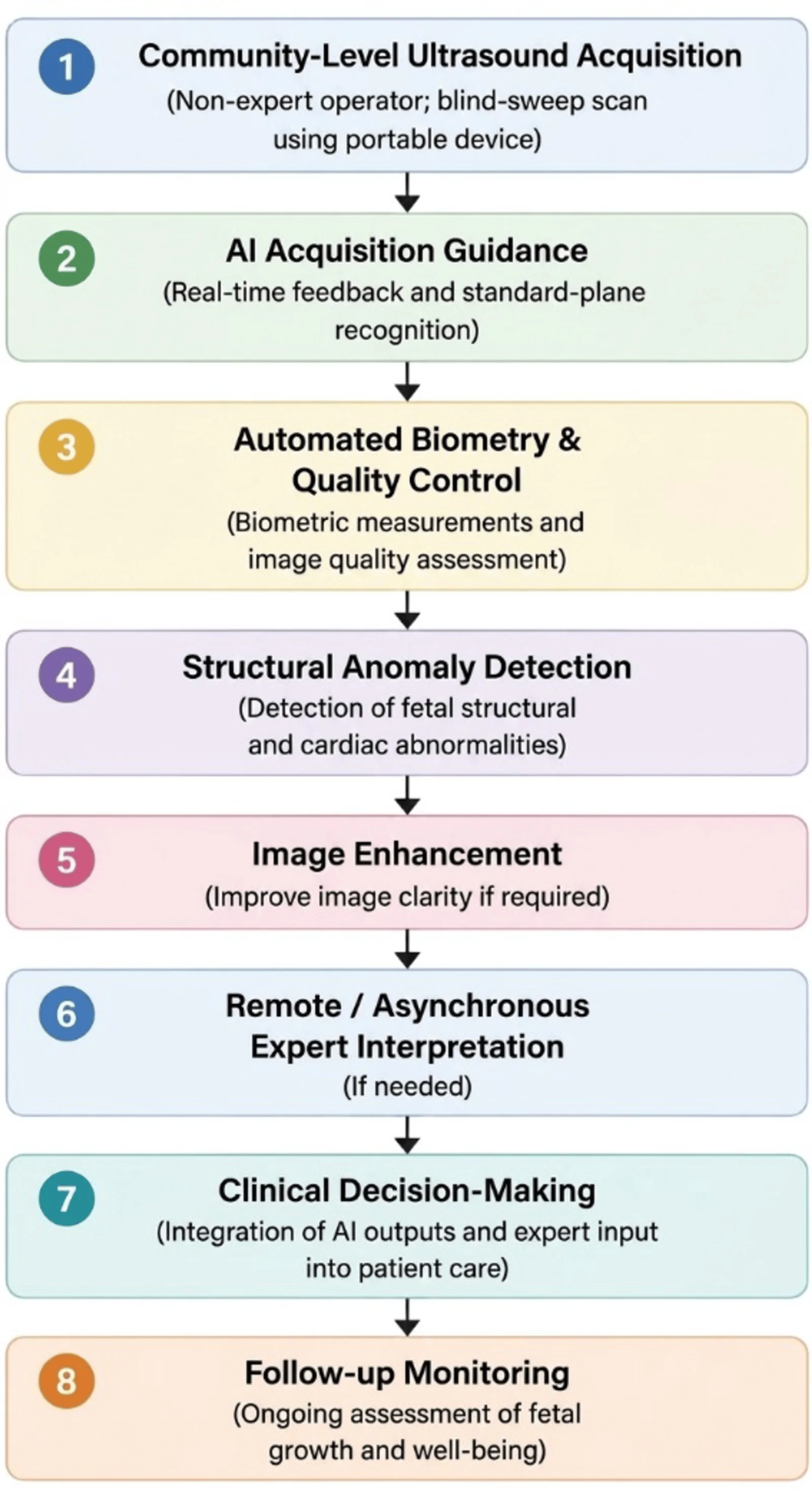
AI-assisted fetal ultrasound care pathway in low-resource settings Original figure created by the authors using https://whimsical.com/ (Whimsical, Denver, Colorado, US)

Neonatal disorders remain among the leading global causes of years of life lost despite substantial reductions in age-standardized mortality rates achieved over the past three decades, with sub-Saharan Africa continuing to bear a markedly lower mean age at death than the global average, reflecting the concentration of perinatal and maternal mortality in this region [5]. Congenital heart defects illustrate this access gap concretely: prenatal detection rates for congenital heart disease vary widely worldwide, from 34% to 85%, with some low- and middle-income countries detecting as few as 9.3% of cases before birth, a shortfall attributed in part to the scarcity of fetal echocardiography expertise outside specialist centers [13]. Systematic review evidence indicates that task-shifting point-of-care ultrasound to non-specialist healthcare workers, including midwives, nurses, and community health workers, can expand diagnostic imaging capacity in primary care settings in low- and middle-income countries (LMICs) and meaningfully affect patient management, but implementation is hindered by the cost of face-to-face training, unreliable internet connectivity for telemedicine-supported interpretation, and unstable electricity supply [4]. These structural constraints frame the rationale for AI-assisted approaches that reduce dependence on continuous expert oversight. The overall AI-assisted fetal ultrasound care pathway in low-resource settings is illustrated in Figure 1.

Applications of artificial intelligence in fetal ultrasound relevant to low-resource settings

The reviewed literature clusters into six functional domains along the fetal ultrasound care pathway, summarized in Table 1.

**Table 1:** Functional domains of AI-assisted fetal ultrasound applications relevant to low-resource settings Created by the authors using information synthesized from the cited references.

Domain	Core Technical Approach	Clinical Objective	Representative studies
Gestational age estimation	Deep learning applied to blind-sweep or non-targeted 2D/video ultrasound acquired by novice operators	Accurate pregnancy dating without expert biometric interpretation	[1,9,12,14,15]
Automated biometry and image quality control	Convolutional neural networks and segmentation models for standard-plane recognition and post-hoc quality scoring	Ensuring diagnostic-grade image acquisition by non-specialist providers	[16-18]
Structural anomaly and growth-disorder detection	Segmentation and classification networks (e.g., SegNet, UNet, EfficientNet) applied to fetal head, cardiac, and biometric structures	Early detection of microcephaly, macrocephaly, congenital heart defects, and growth restriction/macrosomia	[13,19,20]
Fetal cardiac and movement monitoring	Neural sequential modeling of Doppler ultrasound signals; wearable inertial and accelerometer-based sensing	Continuous, low-cost surveillance of fetal well-being outside specialist units	[21-23]
Image enhancement for low-cost hardware	Super-resolution generative and transformer-based models (e.g., Real-ESRGAN, SwinIR)	Compensating for the lower image resolution of low-cost or portable ultrasound probes	[18,24]
Edge and resource-constrained deployment	Knowledge distillation, INT8 quantization, and compact multi-task/few-shot architectures	Enabling on-device inference without dependence on continuous connectivity or cloud infrastructure	[22,25]

I. Gestational Age Estimation From Novice-Acquired Scans

Accurate GA estimation underpins virtually all downstream obstetric decision-making, yet it is often the ultrasound task most dependent on operator skill. The conceptual template for AI-assisted, novice-operator GA estimation was established by van den Heuvel et al., who trained fully convolutional neural networks to automatically detect the fetal head and estimate head circumference from free-hand obstetric sweep protocol scans acquired by minimally trained health workers in Wolisso, Ethiopia, showing that gestational age derived from these automated measurements fell within the expected reference interval for most participants and thereby demonstrating feasibility of the approach in a genuinely resource-constrained setting nearly a decade before larger multicenter validations were undertaken [9]. Building on this foundation, Naz et al. conducted a systematic review and meta-analysis of AI models for GA estimation, pooling 10 studies and reporting a pooled mean error of 4.32 days for models using 2-dimensional images and 2.55 days for models using blind-sweep video, with accuracy varying by trimester and income setting of the source data; notably, only one of the 17 included studies originated from a LMIC, highlighting a persistent representation gap [14].

Benson et al. trained a deep-learning model on more than two million ultrasound images from 78,531 pregnancies across Australia, India, and the United Kingdom to estimate GA from non-targeted fetal ultrasound images regardless of orientation, achieving a mean absolute error of 1.7 days at 14-18 weeks and 2.8 days at 18-24 weeks, outperforming traditional biometry and processing video in a median of 24 seconds [12]. Building directly on the blind-sweep paradigm designed for novice operators, Willis et al. evaluated the cross-setting generalizability of an AI system in a prospective diagnostic study spanning Chicago, USA, and Nairobi, Kenya, reporting a mean absolute error of 4.1-4.3 days after lightweight domain adaptation and demonstrating statistical noninferiority to expert sonographer estimates, with the authors explicitly framing the approach as supporting scalable cross-setting deployment in low-resource settings [1]. The formative PEARLS study protocol in Ghana, Kenya, and South Africa similarly incorporates validation of an AI ultrasound tool for gestational age estimation as a precursor to a pre-eclampsia risk-screening and aspirin-dosing trial, reflecting the operational integration of AI dating tools into LMIC trial infrastructure [15]. The diagnostic performance of representative AI models for gestational age estimation is summarized in Table 2.

**Table 2:** Reported diagnostic performance of AI models for gestational age estimation from fetal ultrasound Created by the authors using information synthesized from the cited references. HIC: high-income country; UMIC: upper-middle-income country; LMIC: low-and middle-income country; MAE: mean absolute error; BPD: biparietal diameter; AC: abdominal circumference; HC: head circumference; FL: femur length

Study	Study Design	Data Source/Setting	Input Modality	Reported Accuracy	Comparator
van den Heuvel et al., 2018 [9]	Prospective, single-center, method development and validation study	Wolisso, Ethiopia (183 pregnant women; obstetric sweep protocol data acquired by an experienced sonographer)	Free-hand obstetric sweep video with automated fetal head detection and circumference estimation	Most automated gestational age estimates fell within the P2.5-P97.5 interval of the Hadlock reference curve	Reference head circumference measured from the standard plane by an experienced sonographer
Naz et al., 2025 [14]	Systematic review and meta-analysis	17 studies (predominantly HICs/UMICs; one LMIC-only study)	2D ultrasound images and blind-sweep videos	Pooled mean error: 4.32 days for 2D images and 2.55 days for blind-sweep videos	Standard obstetric ultrasound fetal biometry (gold standard)
Benson et al., 2025 [12]	Retrospective, multicenter, diagnostic accuracy study	Australia, India, and UK; 2,000,329 images from 78,531 pregnancies (training); 36,762 images from 742 fetuses (validation)	Non-targeted 2D ultrasound images and undirected ultrasound videos (any orientation)	MAE: 1.7 days (14-18 weeks) and 2.8 days (18-24 weeks), significantly outperforming conventional biometry	Conventional fetal biometry (BPD, HC, AC, FL) referenced to first-trimester CRL-based gestational age (gold standard)
Willis et al., 2026 [1]	Prospective, multicenter observational diagnostic study	Chicago, USA and Nairobi, Kenya (385 participants, primary evaluation set)	Blind-sweep ultrasound acquired by novice operators	MAE 4.1 days (Chicago); 4.3 days (Nairobi); pooled MAE 4.2 days; noninferior to the clinical standard (P < 0.001)	Standard clinical gestational age estimate (Hadlock formula-based expert sonographer estimate)

II. Automated Biometry, Quality Control, and Standard-Plane Recognition

Because measurement accuracy depends on acquiring correct anatomical planes, several groups have focused on automating plane recognition and post-hoc quality assurance rather than replacing the sonographer entirely. This line of work has a substantial pre-history: Yaqub et al. described an early deep-learning solution for automatic verification that transventricular fetal neurosonographic planes met clinical standard constraints, evaluated against a large real-world clinical dataset and shown to approach human expert assessment, while Dong et al. subsequently proposed a generic deep-learning framework for automated quality control of fetal cardiac four-chamber views, achieving a mean average precision of 93.52% [8,10]. Ryou et al. extended automated assessment to three-dimensional first-trimester volumes, combining segmentation, plane-orientation estimation, and automated biometry within a single pipeline [11].

More recent large-scale evaluations have tested these principles at health-system scale: Zhao et al. reported a multicenter real-world evaluation of an AI-based quality-control system deployed across 34 hospitals in Guizhou Province, China, analyzing 61,959 examinations comprising 551,144 images over 36 months; the proportion of examinations meeting full-standard criteria rose substantially across all scan categories, for example from 39.5% to 82.1% for first-trimester scans, with sustained improvement associated with system deployment, although the authors cautioned that a direct link between image-quality improvement and perinatal outcome has not yet been established [16]. The Intrapartum Ultrasound Grand Challenge, co-hosted with MICCAI 2024, produced the largest multicenter intrapartum ultrasound video dataset to date (774 videos, 68,106 images from three hospitals) and benchmarked multi-task deep-learning frameworks integrating standard-plane classification, segmentation, and biometry for labor-progress monitoring, concluding that although promising, the field remains at an early stage requiring further methodological maturation before clinical implementation [17]. The AMAL-For-Qatar initiative has similarly assembled one of the largest publicly available annotated datasets for fetal head biometry (3,832 images) alongside a segmentation model (FetSAM) and an automated reporting system, explicitly incorporating super-resolution techniques intended for low-resource deployment [18].

III. Detection of Structural and Growth Abnormalities

Beyond biometry, AI models have been applied to detect specific structural anomalies. Mengistu et al. developed and evaluated a deep-learning pipeline (comparing SegNet, UNet, FCN, MobileNetV2, and EfficientNet-B0 architectures) for automatic segmentation and classification of microcephaly and macrocephaly using ultrasound data collected from three Ethiopian healthcare facilities, reporting an accuracy of 98% and Dice coefficient of 0.97 for segmentation, with measurement accuracies of 92.5% and 91.2% relative to industry experts for biparietal diameter and head circumference, respectively, explicitly framing the work around Ethiopia’s shortage of trained personnel and diagnostic machines [19]. The international multicenter CAIFE (Clinical Artificial Intelligence in Fetal Echocardiography) study protocol aims to curate over 16,000 retrospective and prospective ultrasound scans to build models differentiating normal fetal hearts from those with congenital heart defects, with an explicit objective of supporting detection “particularly in nonspecialist or low-resource settings where fetal echocardiography expertise is not readily available” [13]. A comprehensive review by Ługowski et al. of AI applications in fetal growth restriction, small-for-gestational-age, large-for-gestational-age, and macrosomia concluded that models integrating maternal, fetal, and imaging data achieve accuracy comparable to experienced clinicians while enhancing efficiency, though most published studies remain limited by retrospective design, small sample sizes, and absent external validation [20].

IV. Fetal Cardiac and Movement Monitoring

Continuous fetal surveillance outside specialist units has been targeted through both Doppler signal processing and wearable sensing. Rafiei et al. introduced AutoFHR, an interpretable neural sequential model using dilated causal convolutions and attention mechanisms to estimate fetal heart rate from one-dimensional Doppler ultrasound signals, a modality selected specifically for its affordability, portability, and simplicity in low-resource settings; the model achieved root-mean-square errors of 2.2-2.8 beats per minute across development and external validation datasets, outperforming conventional rigid algorithms [21]. Complementing this, Rattanasak et al. developed a lightweight deep-learning framework for fetal movement monitoring deployable on resource-constrained microcontrollers via knowledge distillation and quantization, reducing memory footprint by 74.8% while achieving a sensitivity of 90.05% and enabling roughly 25 hours of continuous battery-powered operation, a design explicitly intended to extend prenatal monitoring capability into low-resource contexts [22]. A registered systematic review protocol is further synthesizing evidence on wearable devices for antenatal fetal monitoring more broadly, noting the potential of such technologies to enable continuous, real-time data collection outside clinical environments in resource-limited settings [23].

V. Image Enhancement and Edge Deployment

Low-cost ultrasound probes used in LRS often produce images of lower resolution than tertiary-center equipment, and Boumeridja et al. systematically evaluated five super-resolution techniques (dual back-projection based internal learning (DBPISR), blind super-resolution generative adversarial network (BSRGAN), real-world enhanced super-resolution generative adversarial network (Real-ESRGAN), swin transformer for image restoration (SwinIR), and SwinIR-Large) applied to fetal ultrasound images sourced from five developing countries, finding that Real-ESRGAN consistently improved both image quality metrics and downstream anatomical plane classification accuracy even with limited and variable datasets, supporting super-resolution as a practical strategy to offset hardware constraints [24]. Complementary work on compact multimodal architectures, including Siamese networks with few-shot learning and post-training INT8 quantization to reduce model size below 10 MB, has likewise been oriented toward enabling edge deployment for prenatal anomaly detection where cloud connectivity cannot be assumed [25].

VI. Real-World Implementation Experience

Evidence of deployment beyond controlled research settings remains comparatively scarce, but a small number of studies provide early real-world implementation data. Sesay et al. conducted a mixed-methods evaluation of an AI-enabled smartphone-based obstetric ultrasound device piloted in Tonkolili District, Sierra Leone, analyzing 2,315 obstetric sweep protocols performed by 81 healthcare providers across seven facilities; 83.8% of sweeps were of sufficient quality for remote or AI analysis, tutorial videos and provider skill level significantly predicted scan quality, and the device was well accepted by both providers and clients, although infrastructure constraints, supply-chain limitations, absent comprehensive guidelines, and funding gaps were identified as barriers to broader scale-up using the Non-adoption, Abandonment, Scale-up, Spread, and Sustainability (NASSS) framework [3]. In Uganda, a cross-sectional pilot protocol is establishing one of the first annotated obstetric Doppler ultrasound datasets derived entirely from an African population to train machine-learning models predicting pre-eclampsia complications, explicitly framed as addressing global gaps in representation in obstetric AI datasets [26].

Opportunities

The reviewed literature converges on several recurring opportunities. First, AI-guided blind-sweep acquisition protocols lower the skill threshold for image capture, enabling task-shifting to midwives and community health workers, consistent with prior evidence that task-shifted point-of-care ultrasound is feasible and clinically impactful in primary-care LMIC settings [1,4]. Second, automated quality control provides a scalable substitute for on-site expert supervision, as demonstrated by sustained, multi-year improvements in standardized image acquisition across an entire provincial health system in China [16]. Third, model architectures optimized for computational efficiency, including knowledge distillation, quantization, and super-resolution preprocessing, directly address the hardware and connectivity constraints characteristic of LRS, enabling edge deployment without dependence on continuous internet access [22,24,25]. Fourth, several initiatives are deliberately generating training and validation data from underrepresented populations in sub-Saharan Africa, addressing a long-standing gap in which the large training datasets underpinning commercial AI ultrasound tools have been drawn predominantly from high-income settings [1,19,26].

Challenges

Despite these opportunities, substantial challenges temper enthusiasm for near-term widespread adoption. The major barriers to implementing AI-assisted fetal ultrasound in low-resource settings, together with potential enabling strategies, are summarized in Table 3. Representation and generalizability remain central concerns: the meta-analysis by Naz et al. found that only a small minority of included studies originated from low-income countries, with most pooled evidence drawn from high- and upper-middle-income settings, raising the possibility that reported accuracy metrics do not generalize to the populations, fetal genetics, maternal body habitus distributions, and equipment types most relevant to LRS [14]. Even where cross-setting generalization has been formally tested, as in the Chicago-Nairobi comparison, performance required lightweight model adaptation using local data, indicating that “out-of-the-box” deployment across geographically and technologically diverse settings cannot be assumed [1]. Methodological quality is a further limitation: reviews of AI applications in fetal growth disorders and in broader perinatal care consistently note that most published studies are retrospective, single-center, and lack external validation, with low-resource settings, especially sub-Saharan Africa, remaining underrepresented and few solutions integrated into electronic health records [20,27].

**Table 3:** Key barriers to implementation of AI-assisted fetal ultrasound in low-resource settings and corresponding enabling strategies Sources: [1,3,14,16,17,20,27,28] Created by the authors using information synthesized from the cited references.

Barrier Category	Specific Challenge Identified	Enabling Strategy Identified in Reviewed Literature
Data representativeness	Training and validation datasets drawn predominantly from high- and upper-middle-income settings; sub-Saharan Africa underrepresented	Prospective generation of locally sourced, annotated datasets (e.g., African Doppler and biometry cohorts) and cross-setting adaptation studies
Infrastructure and connectivity	Unreliable electricity, poor internet connectivity limiting telemedicine-supported interpretation, hardware supply-chain constraints	Edge-deployable, quantized, and compressed models not dependent on continuous connectivity; offline-capable smartphone-based devices
Image quality/hardware limitations	Low-cost portable probes produce lower-resolution images than tertiary-center equipment	Super-resolution preprocessing to enhance image quality prior to interpretation or classification
Evidence quality and validation	Predominantly retrospective, single-center studies with limited external validation; unclear linkage of image-quality metrics to clinical outcomes	Prospective multicenter and cross-country validation studies with noninferiority designs against expert reference standards
Workforce and training	High cost of face-to-face training for non-specialist operators; variable scan quality dependent on operator skill	Structured tutorial-video-based training protocols; blind-sweep acquisition designed to be operator-agnostic
Governance and regulation	Absence of comprehensive operational guidelines; unresolved data-privacy, liability, and algorithmic-bias considerations	Development of governance and regulatory frameworks specific to AI diagnostic tools used by non-specialist providers

Clinical benefit beyond process metrics is also incompletely demonstrated; even the large-scale Guizhou quality-control study, despite showing marked and sustained gains in image standardization, explicitly stated that linkage between image quality improvement and diagnostic performance or perinatal outcomes required further study [16]. Infrastructural barriers identified in the Sierra Leone implementation study, including unreliable connectivity, supply chain constraints, absent operational guidelines, and funding gaps, illustrate that technical accuracy alone is insufficient for sustainable scale-up, and that system-level factors captured by implementation science frameworks, such as NASSS, must be addressed concurrently [3]. Finally, the IUGC (Intrapartum Ultrasound Grand Challenge) challenge investigators, despite assembling the largest available multicenter intrapartum ultrasound dataset and evaluating eight competing algorithmic approaches, concluded that the field remains in an early stage of maturity, underscoring that even well-resourced, collaborative benchmarking exercises have not yet produced solutions ready for routine clinical deployment [17]. Data privacy, algorithmic bias, regulatory approval pathways, and clinician trust are additionally flagged as unresolved considerations across multiple reviews spanning obstetric and broader perinatal AI applications [27,28].

Future directions

Several priorities emerge from this synthesis. Prospective, multicenter validation studies conducted directly within LRS, rather than retrospective external application of models trained elsewhere, are needed to establish generalizable accuracy estimates; the Chicago-Nairobi and West China multicenter studies offer methodological templates that could be extended to a broader range of LMIC settings [1,16]. Linkage of image-quality or diagnostic-accuracy endpoints to downstream perinatal outcomes, rather than technical performance metrics alone, should be prioritized, as explicitly recommended by the authors of the Guizhou quality-control study [16]. Continued investment in computationally efficient, edge-deployable architectures, including quantized networks and super-resolution preprocessing, will remain important given the persistence of connectivity and hardware constraints in many LRS [22,24,25].

Dataset diversification efforts, such as the Uganda pre-eclampsia Doppler dataset and the Ethiopian microcephaly/macrocephaly cohort, should be expanded and, where possible, harmonized into shared, ethically governed repositories to support external validation across African, South Asian, and Latin American populations [19,26]. Given the persistent scarcity of expert-annotated training data in LRS, weakly supervised and self-supervised representation-learning approaches, which reduce dependence on large volumes of manually labeled images by learning transferable features from raw or minimally labeled ultrasound video, warrant continued investment as a means of lowering the annotation burden associated with model development in these settings [29-31]. Implementation science frameworks should be embedded prospectively into deployment studies from their inception, rather than applied retrospectively, to systematically characterize adoption barriers as was done in the Sierra Leone pilot [3]. Finally, regulatory and governance frameworks specific to AI-based diagnostic tools intended for use by non-specialist providers in LRS are needed, addressing liability, data protection, and standards for acceptable diagnostic performance thresholds, an area flagged as underdeveloped across the reviewed literature [18,27].

## Conclusions

Artificial intelligence-assisted fetal ultrasound has the potential to transform antenatal imaging in low-resource settings by improving access to timely and reliable prenatal assessment despite shortages of skilled sonographers. Current evidence demonstrates promising performance across a range of applications, including gestational age estimation, automated biometry, image quality assessment, and detection of selected fetal structural abnormalities. Nevertheless, most available studies are retrospective, conducted in relatively well-resourced settings, and provide limited evidence regarding long-term clinical effectiveness and implementation in routine practice within low-resource environments.

Future research should prioritize prospective multicenter validation in diverse low-resource populations, development of locally representative datasets, and robust regulatory and governance frameworks to ensure safe and equitable deployment. Equally important, comprehensive health economic evaluations, including cost-effectiveness and budget impact analyses, are needed to determine whether AI-assisted fetal ultrasound represents a sustainable and affordable investment for resource-constrained healthcare systems. Addressing these clinical, technical, economic, and implementation challenges will be essential to translate promising AI innovations into scalable solutions that improve maternal and fetal health outcomes in underserved populations.
